# Heat-Labile Enterotoxin-Induced PERK-CHOP Pathway Activation Causes Intestinal Epithelial Cell Apoptosis

**DOI:** 10.3389/fcimb.2017.00244

**Published:** 2017-06-08

**Authors:** Xi Lu, Chunmeng Li, Congcong Li, Pengcheng Li, Enqing Fu, Yonghong Xie, Faguang Jin

**Affiliations:** ^1^Department of Respiration, Tangdu Hospital, Fourth Military Medical UniversityXi'an, China; ^2^Bacteriology Room in Department of Clinical Laboratory, Shaanxi Province Hospital of Traditional Chinese MedicineXi'an, China

**Keywords:** enterotoxigenic *Escherichia coli*, heat-labile enterotoxin, apoptosis, endoplasmic reticulum stress, ROS

## Abstract

Enterotoxigenic *Escherichia coli* (ETEC) is a leading cause of diarrhea among children and travelers in developing countries, and heat-labile enterotoxin (LT) is one of the most important virulence factors. The pathogenesis of and virulence factors associated with ETEC have been well-characterized; however, the extent to which ETEC damages host cells remains unclear. In this study, we found that LT could induce decreases in intestinal epithelial cell viability and induce apoptosis in a dose- and time- dependent manner in both HCT-8 and Caco-2 cells. We analyzed the expression profiles of apoptosis-related proteins via protein array technology and found that Bax, p-p53(S46), cleaved caspase-3, and TNFRI/TNFRSF1A expression levels were significantly up-regulated in wild-type ETEC- but not in ΔLT ETEC-infected HCT-8 cells. Bax is essential for endoplasmic reticulum (ER) stress-triggered apoptosis, and our RNAi experiments showed that the PERK-eIF2-CHOP pathway and reactive oxygen species (ROS) are also main participants in this process. LT-induced ROS generation was decreased in CHOP-knockdown HCT-8 cells compared to that in control cells. Moreover, pretreatment with the ROS inhibitor NAC down-regulated GRP78, CHOP, Bim, and cleaved caspase-3 expression, resulting in a reduction in the apoptosis rate from 36.2 to 20.3% in LT-treated HCT-8 cells. Furthermore, ROS inhibition also attenuated LT-induced apoptosis in the small intestinal mucosa in the ETEC-inoculation mouse model.

## Introduction

Enterotoxigenic *Escherichia coli* (ETEC) is an important pathogen that causes human and porcine morbidity and mortality (Crossman et al., 2010). Worldwide, ETEC is responsible for 200 million infections annually and is a leading cause of mortality due to infectious diarrhea in young children in developing countries (Gupta et al., 2008). ETEC produces several virulence factors, including colonization factors (CFs) that are responsible for facilitating cell adhesion to the host small intestinal epithelium, and heat-stable (ST), and heat-labile enterotoxins (LTs) that induce a net secretory state leading to profuse watery diarrhea.

The molecular basis of ETEC enterotoxin expression in the host environment is becoming clearer. Studies have shown that enterotoxin expression is sensitive to Na^+^ and glucose, which ETEC encounters upon host cell attachment, and that the transcriptional response of ETEC to glucose is controlled by cAMP receptor protein, which serves as a virulence regulator (Bodero and Munson, 2009; Haycocks et al., 2015). In addition, terminal electron acceptors, which serve as metabolites in the intestine, can activate heat-labile enterotoxin (LT) secretion under intestinal anaerobic conditions by promoting GspD assembly (Lu et al., 2016). However, research on the pathogenic mechanism underlying the effects of the enterotoxin on the host has also deepened the understanding of the processes by which the enterotoxin interacts with the host. These studies have shown that LT can not only subvert innate immune responses by blocking host NF-κB activation (Wang and Hardwidge, 2012) but also enhance ETEC adherence by activating the MAPK pathway in intestinal epithelial cells (Johnson et al., 2009; Wang et al., 2012).

Some pathogens have the ability to turn some host protective functions against the host. The reactive oxygen species (ROS) produced by phagocytes (Rokutan et al., 2006) or intestinal epithelial cells via Nox1 family proteins (Hartog et al., 2016) can kill invasive bacteria; however, prolonged unchecked ROS generation has been implicated in host cell DNA damage and chronic disease development and has even been shown to lead to cancer (e.g., *Helicobacter pylori*) (Jenks et al., 2003; Nardone et al., 2004). Endoplasmic reticulum (ER) stress is also a double-edge sword. The ER stress response can promote cellular repair and sustained survival by reducing the unfolded protein load through global attenuation of protein synthesis and/or up-regulation of chaperone enzymes and ER structural components (Malhotra and Kaufman, 2007). However, when ER stress is prolonged or the adaptive response fails, cells undergo apoptosis (Puthalakath et al., 2007). The following three independent ER stress receptors mediate the unfolded protein response (UPR): pancreatic ER kinase-like ER kinase (PERK), activating transcription factor 6 (ATF6), and inositol-requiring enzyme 1 (IRE1), all of which are activated by GRP78 dissociation (Szegezdi et al., 2006).

The pathogenesis of and virulence factors associated with ETEC have been well-characterized; however, the extent to which ETEC damages host cells remains unclear (Tang et al., 2014). To explore the subtle relationship between ETEC enterotoxin and the host further, we investigated the apoptotic effects of LT exposure on intestinal epithelial cells, as well as the signaling pathways that may be responsible for mediating these effects. We found that LT can induce intestinal epithelial cell apoptosis through the ER stress signaling pathway. We also examined the activation states of the three receptors that mediate the UPR under LT stimulation and assessed the contributions of each pathway to LT-induced intestinal epithelial cell apoptosis.

## Materials and methods

### Cell culture

HCT-8 and Caco-2 cells are maintained at 37°C in 5% CO_2_ in Dulbecco's modified Eagle's medium (DMEM; Hyclone, USA) supplemented with 10% fetal bovine serum (FBS; Sijiqing China). Cells in exponential growth phase were used for all experiments.

### Oligonucleotides, bacterial strains, plasmids, and molecular biology experiments

The bacteria, plasmids, strains and oligonucleotides used in this study are shown in Tables 1, 2. PCR was used to amplify the genes encoding the enterotoxin subunits LT (*eltAB*), LT-A (*eltA*), LT-B (*eltB*), and STa1 (e*stA1*) from ETEC H10407 (Evans and Dupont, 1977), and the *estB* gene, which encodes STb, from ETEC TD2385 genomic DNA, using the primers ltAB-1/ltAB-2, ltA-1/ltAB2, ltAB-1/ltB-2, sta1-1/sta2, and stb-1/stb-2, respectively (Table 1), via *Xho*I and *Hind*III restriction digestion. The products were then cloned into a pBAD/His vector and transferred into G58-1 cells (Francis and Willgohs, 1991; Dorsey et al., 2006; Johnson et al., 2009), which can be expressed under L-arabinose induction. LT was extracted from *E. coli* k12 containing the pEWD299 plasmid (Dallas et al., 1979) and was purified by one-step chromatography with an immobilized D-galactose column, as previously described (Uesaka et al., 1994). The purity of LT in the resulting fractions was determined by SDS-PAGE and high-performance liquid chromatography (HPLC), the concentration was determined by GM1-ELISA (Wijemanne et al., 2015), and western blotting was used for the qualitative detection of LT toxin. The fractions were stored at −80°C until use. Overlap extension-PCR (Warrens et al., 1997) was used to construct LT A72R site-directed mutants using the primers A72R-f and A72R-r (Johnson et al., 2009).

**Table 1:** Strains and plasmids used in this study.

**Strains or plasmids**	**Relevant phenotype/genotype and description**	**Source or references**
**STRAINS**		
ETEC H10407	Wild-type ETEC serotype O78:H11,LT^+^ ST^+^	Evans and Dupont, 1977
ETEC H10407 ΔLT	H10407 derivative with deletion in *eltAB*	Dorsey et al., 2006
*E. coli* G58-1	WT non-toxigenic *E. coli* strain of prorcine origin O101:K28:NM, LT^−^, STb^−^	Francis and Willgohs, 1991
*E. coli* C600	*E. coli* K-12 containing pEWD299	Dallas et al., 1979
**PLASMIDS**		
pEWD299	A derivative plasmid from pEWD022 containing LT holotoxin gene *eltAB*, Amp^r^, Tet^r^	Dallas et al., 1979
pEWD501	A derivative plasmid from pEWD299 without *eltAB*, Amp^r^, Tet^r^	Dallas et al., 1979
pBAD	Amp^*r*^; araC; P_*BAD*_ promoter 6 × His, MSC	Invitrogen
pBAD-LT	LT holotoxin gene *eltAB* cloned between *Xho*I and *Hind*III of pBAD	This study
pBAD-LT-A	LT-A subunit gene *eltA* cloned between *Xho*I and *Hind*III of pBAD	This study
pBAD-LT-B	LT-B subunit gene *eltB* cloned between *Xho*I and *Hind*III of pBAD	This study
pBAD-STa	STa gene *sta1* cloned between *Xho*I and *Hind*III of pBAD	This study
pBAD-STb	STb gene *stb* cloned between *Xho*I and *Hind*III of pBAD	This study
pBAD-LT(A72R)	LT holotoxin gene A72R site mutant cloned between *XhoI* and *HindIII* of pBAD	This study

**Table 2:** Primers used in this study.

**Primer name**	**Sequence(5′–3′)**	**Description/application**	**Source or references**
ltAB-1	**CTCGAG**CTAGTTTTCCATACTGATTGCCGCA	P*bad*-ltAB & P*bad*-ltB clone	This study
ltAB-2	**TTCGAA**ATGAAAAATATAACTTTCATT	P*bad*-ltAB & P*bad*-ltA clone	This study
ltA-1	**CTCGAG**TCATAATTCATTCCGAATTCTGT	P*bad*-ltA clone	This study
ltB-2	**TTCGAA**ATGAATAAAGTAAAATGTTATGTTT	P*bad*-ltB clone	This study
sta1-1	**CTCGAG**TTAATAACATCCAGCACAGGCAGGA	P*bad*-stA1clone	This study
sta1-2	**TTCGAA**ATGAAAAAGCTAATGTTGGCAATTT		This study
stb-1	**CTCGAG**TATTATATTTCGAAGCTTAAGTATT	P*bad*-stB clone	This study
stb-2	**TTCGAA**CATGACACGAAGCGCAGGCTGTTGC		This study
A72R-f	GAAGTGCTCACTTACGTGGACAGTCTATATTATCAGG	LT A72R construction	Johnson et al., 2009
A72R-r	CCTGATAATATAGACTGTCCACGTAAGTGAGCACTTC		
XBP1-F	AAACAGAGTAGCAGCTCAGACTGC	For XBP-1 analysis	Lee et al., 2008
XBP1-R	ATCTCTAAGACTAGGGGCTTGGT		

*Bold indicates the restriction enzyme cutting site*.

### Bacterial infections and LT treatment

We grew ETEC strains in CAYE broth overnight at 37°C and then diluted the cultures 1:50 in serum- and antibiotic-free DMEM, in which they were incubated for 3 h to facilitate additional growth. The cell medium was replaced with serum- and antibiotic-free DMEM before the infections, during which we infected HCT-8 and Caco-2 cells with ETEC at a multiplicity of infection (MOI) of 1:100 for 4 h, unless otherwise indicated. The cells were then washed with cold PBS and stored for subsequent analyses.

HCT-8 or Caco-2 cells were seeded in 24-well plates or 10-cm dishes. When the cells reached ~70% confluence, they were washed with PBS and pretreated with or without ROS, ER stress inhibitors [5 mM NAC, 2 mM 4-phenylbutyric acid (4-PBA)] or activators [150 nM thapsigargin (Thap)] for 2 h before being treated with 0–500 ng/ml LT for 2–24 h.

### Cell viability assay

Cell viability was determined using the Cell Counting Kit-8 (CCK-8) (Seven-Sea Biotech, China), according to the manufacturer's instructions. Intestinal epithelial cells, HCT-8 cells or Caco-2 cells were plated in three replicates in 96-well plates (100 μl, 2^*^10^3^/well). After plating (24 h), the cells were subjected to LT treatment with different concentrations and times. The CCK-8 solution (10 μl) was added to each well, and the cells were incubated for an additional 3 h at 37°C, after which the optical density (OD) values were assessed at 450 nm using a microplate absorbance reader (Thermo Multiskan GO). Cell viability was expressed as the percentage compared with the control cells. All experiments were repeated three times.

### Assessment of intestinal epithelial cell apoptosis

Annexin V-TITC/PI staining was used to quantify the apoptotic effect of LT on HCT-8 or Caco-2 cells with the Annexin V-FITC Apoptosis Detection kit according to the manufacturer's protocol and quantified using flow cytometry. Briefly, cells were cultured overnight in 6-well plates and then exposed to LT treatments or ETEC infection as indicated above. The detached and adherent cells were collected and washed twice with PBS. The cells were then re-suspended in binding buffer and incubated with Annexin V-FITC and propidium iodide (BD Biosciences, USA) to achieved double staining, according to the manufacturer's instructions. The mixture was incubated in the dark for 15 min at room temperature prior to flow cytometry analysis. The terminal dUTP transferase nick end labeling (TUNEL) assay was also applied to detect apoptosis in intestinal epithelial cells. Cells were grown on glass cover slides in 24-well plates and incubated overnight at 37°C. After treatment with LT toxin, the cells were fixed in 4% paraformaldehyde for 30 min at room temperature. After rising with PBS, the cells were permeabilized with 0.1% Triton X-100 in 0.1% sodium citrate for 2 min on ice and then incubated with the TUNEL reagent for 1 h at 37°C in the dark. The cells were subsequently rinsed twice with PBS and stained with 1 μg/ml DAPI for nuclear staining.

### Human apoptosis array kit/proteome profiler™

To analyze the expression profiles of apoptosis-related proteins, we used an Apoptosis Array Kit (R&D Systems) and performed assays with 35 antibodies specific for apoptosis-related proteins. These proteins were subsequently blotted on nitrocellulose membranes in duplicate. Briefly, 1^*^10^7^ cells were harvested, and whole-cell lysates were extracted, according to manufacturer's instructions. Then, 400 μg of protein was mixed with 15 μl of biotinylated antibodies. After pretreatment, the samples were incubated with the assay membranes overnight at 4°C. After the membranes were washed, they were treated sequentially with streptavidin-HRP and chemiluminescent detection reagents. After 2 min, the signals on the X-ray film were quantified by a transmission-mode scanner, and the array images were analyzed using Lane 1D (Sage, China). The average background signal was eliminated, and the arrays were calibrated according to the signal strength of the positive controls. The average signal (integrated pixel density) for each apoptosis-related protein was determined, and the corresponding signals for each protein in different arrays were compared.

### ROS measurements

Changes in ROS levels were detected with 2', 7'-dichlorofluorescin diacetate (DCFH-DA) (Purchased from Nanjing Jiancheng Bioengineering Institue, China). Cells were grown on glass cover slides in 24-well plate and exposed to various treatments as indicated. The cells were stained with 10 μM DCFH-DA for 20 min at 37°C in the dark. After two washes with PBS, the cells were analyzed via fluorescence microscopy (Leica DM4000B). The images were merged using LAS V3.8 software (Leica). Cells showing green fluorescence were considered to be ROS positive. The mean fluorescence density indicating ROS generation was measured using flow cytometry.

### Western blot analysis

The cells were lysed on ice in lysis buffer (Beyotime, Hangzhou, China) according to the manufacturer's instructions, and the lysates were centrifuged at 14,000*g at 4°C, for 15 min. The protein concentration was determined using the BCA Protein Assay Kit (Thermo. USA). Equal amounts (25 μg/lane) of total protein were subjected to electrophoresis in a 10% SDS-polyacrylamide gel. Following electrophoresis, the proteins were electro-transferred to polyvinylidene difluoride membranes (Millipore, USA). The membranes were then blocked with 5% skim milk in TBST at room temperature for 2 h and subsequently incubated with primary antibodies (1:500 to 1:1,000) at 4°C overnight. Anti-78-kDa glucose-regulated protein (GRP78), anti-CHOP, anti-caspase-3, anti-cleaved caspase-3, anti-PERK, anti-phospho-PERK, anti-eukaryotic translation initiation factor 2a (eIF2a), anti-phospho-eIF2a (phospho S51), anti-BIM, and anti-BAX antibodies were obtained from Cell Signaling Technology (Cell Signaling Technology, USA). An anti-β-actin antibody was obtained from Santa Cruz Biotechnology (Santa Cruz Biotechnology, USA). Anti-cleaved transcription factor 6 (ATF6), anti-inositol-requiring enzyme 1a (IRE1a), and anti-phospho-IRE1a (S724) anti-LT subunit (A+B) antibodies were purchased from Abcam (Abcam, USA). The membranes were next washed three times in TBST and incubated with horseradish peroxidase-conjugated goat anti-mouse or anti-rabbit IgG (Santa Cruz Biotechnology, USA) (diluted 1: 5,000) for 1 h. The immune complexes were visualized via fluorography using an enhanced ECL system (Millipore, USA).

### Reverse transcriptase PCR

Cells were harvested at various time points after treatment with LT (50 ng/ml). For all treatments, total RNA was extracted with RNAiso Plus (TaKaRa, Japan). First-strand cDNA was synthesized using random primers with PrimeScript RT reagent Kit (TaKaRa, Japan). All of the steps were conducted according to the manufacturer's instructions. For XBP-1 quantitative detection, the primers were listed in Table 2. The reaction conditions consisted of the following steps: 95°C for 5 min, 95°C for 1 min, 57°C for 30 s, 72°C for 30 s, and 72°C for 5 min, with 32 amplification cycles. The PCR products were analyzed based on (Lee et al., 2008).

### Gene silencing using small interfering RNAs (siRNAs)

siRNAs for CHOP, PERK, and ATF-6α, as well as a non-targeting control siRNA, were purchased from TaKaRa. Transfection was performed using Xfect RNA Transfection Reagent, according to the manufacturer's protocol. Prior to transfection, the cells were seeded in 6-well plates and grown to 80–90% confluence. A total of 100 pmol of siRNAs were diluted in 120 μl of Xfect Reaction Buffer, after which 120 μl of Xfect Reaction Buffer and 10 μl of Xfect RNA Transfection Polymer were mixed together. The mixture was then vortexed for 5 s and incubated for 10 min at room temperature to allow nanoparticle complexes to form. The entire nanoparticle complex solution was then added dropwise to the cell culture medium, and the plate containing the medium was rocked gently to allow the solution to mix. The plate was then incubated for 4 h at 37°C in an atmosphere containing 5% CO_2_, after which fresh media were added to the plate. After transfection, the plate was incubated for 24 h at 37°C in an incubator containing 5% CO_2_.

### Inoculation of mice *In vivo*

This study was performed according to the guidelines of the Laboratory Animal Ethical Commission of the Chinese Academy of Sciences. The ETEC infection model was established according to methods described elsewhere (Allen et al., 2006). Six-week-old female ICR (Institute for Cancer Research) mice were purchased from the Laboratory Animal Center of the Fourth Military Medical University (Xi'an, China). Before oral inoculation, all the mice were treated with streptomycin sulfate and cimetidine, according to the methods devised by Allen et al. (2006), to eliminate the normal bacterial flora of the intestinal tract. The mice were randomly assigned to ETEC inoculation (10 mice) or control groups (5 mice). Five mice in the ETEC inoculation group were pretreated with intraperitoneal injections of NAC (1000 mg/kg body weight; Quadrilatero and Hoffman-Goetz, 2005) in 0.1 ml of PBS, and the other five mice in the ETEC inoculation group, as well as the five mice in the control group, were injected intraperitoneally with only 0.1 ml of PBS. The mice in ETEC inoculation group were then orally inoculated with a suspension of ETEC (2*10^7^ cells). At 24 h post-inoculation, all of the mice in each group were sacrificed by cervical dislocation. Subsequently, a fixative solution of 4% paraformaldehyde was injected into the intestinal lumen immediately, and the entire small intestine was then removed and put into the same fixative solution for 24 h and imbedded in paraffin. The fixation procedure was completed within half an hour after euthanasia. The samples were cut into 5-μm-thick transverse sections, and every 10th section was collected and stained with hematoxylin and eosin (HE) or TUNEL staining. For villus height measurement, 10 villi with lamina propria were selected per section. The villus length was measured from the villus tip to the bottom, not including the intestinal crypt. The average length of 10 villi per section indicated the mean villus height for each section. The average height of 10 sections per mouse indicated the mean villus height for each mouse. The mean villus heights of five mice in each group were used to represent the mean villus height for each group.

### Statistical methods

The results of the experiments were expressed as the mean ± *SD*, and ANOVAs or *t*-tests were used to compare the experimental data. The level of significance was considered at a *P* < 0.05.

### Ethics statement

All animal work was performed according to the guidelines of the Laboratory Animal Ethical Commission of the Chinese Academy of Sciences, and protocols were approved by the Institutional Animal Ethics Committee of Tangdu Hospital the Fourth Military Medical University, Xi'an, China (TDLL-2014138, revised 2014).

## Results

### LT induces cell growth inhibition and apoptosis in intestinal epithelial cells

**Figure 1:**
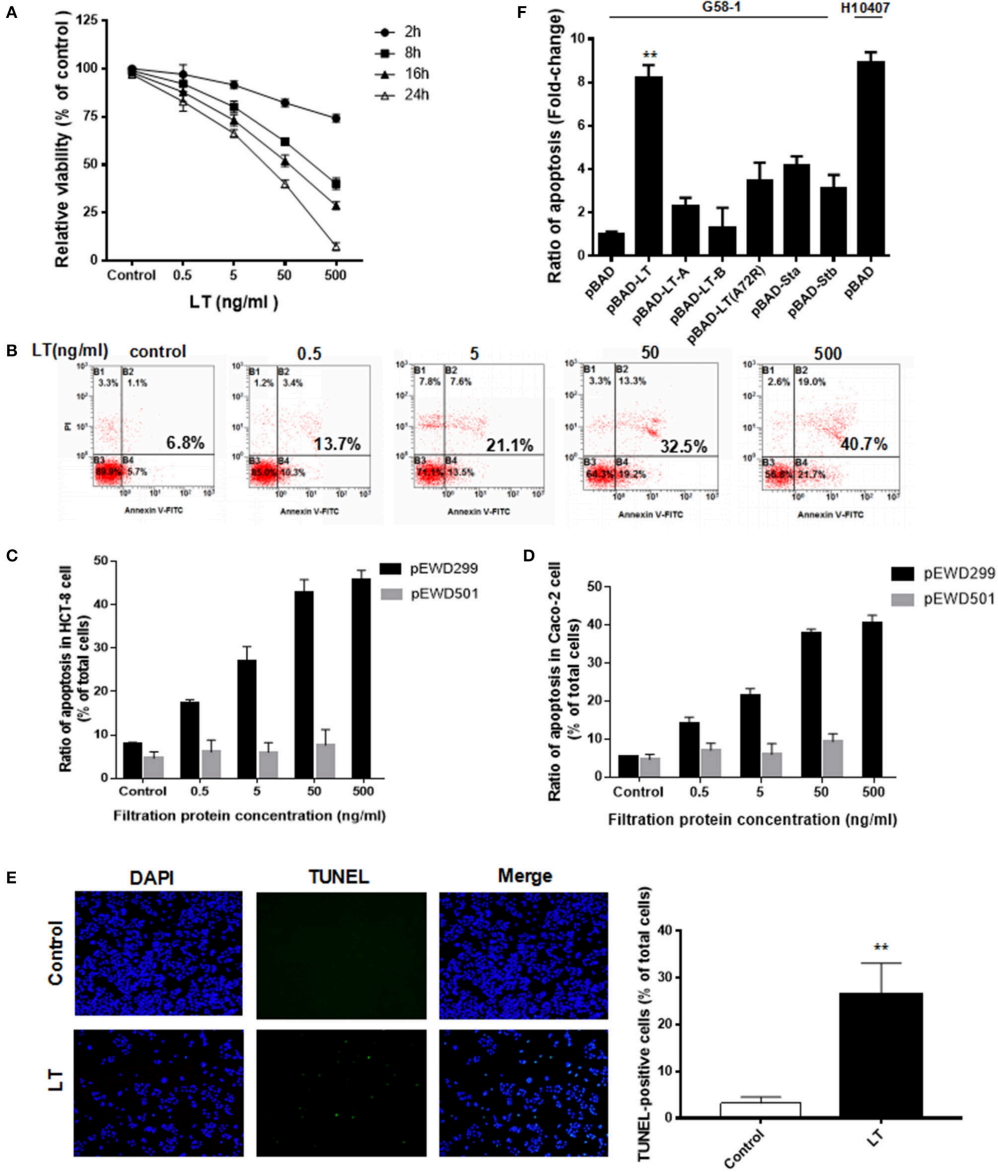
LT inhibits cell growth and induces apoptosis in intestinal epithelial cells. **(A)** The dose- and time-dependent effects of LT on HCT-8 cell viability in cells treated with different concentrations of LT (0, 0.5, 5, 50, 500 ng/ml) for 2, 8, 16, and 24 h were assessed by CCK-8 assay. The TEAN buffer was used as a control. **(B)** The dose-dependent effects of LT on cell apoptosis in cells treated with the indicated concentrations of LT for 16 h were assessed using Annexin V-FITC/PI double-staining and quantified via flow cytometry analysis. The cells shown in the bottom right quadrant were Annexin V-FITC positive and propidium iodide negative. Thus, they were in an early stage of apoptosis. The cells in the top right quadrant stained positively for Annexin V-FITC and propidium iodide. Thus, they were secondary late apoptotic/necrotic cells. The total percentage of cell death is shown in bold. **(C)** The apoptotic effects in HCT-8 cells treated with the different concentrations of purified protein lysates from *E. coli* K12 containing pEWD299 (with the *eltAB* gene) and pEWD501 (a derivative plasmid from pEWD299 without the *eltAB* gene) for 16 h were assessed by Annexin V-FITC/PI double-staining and quantified via flow cytometry analysis. The purification steps were performed in accordance with the LT purification method. The TEAN buffer was used as a control. **(D)** The apoptotic effects in Caco-2 cells treated the same way as C. **(E)** HCT-8 cells were stained with TUNEL after being treated with 50 ng/ml LT for 16 h, and then subsequently observed under a fluorescence microscope. The nuclei are shown in blue, and the TUNEL-positive cells are shown in green. **(F)** The effects of different holotoxin and toxin subunits on HCT-8 cell apoptosis. The LT holotoxin and its subunits, as well as STa1 and STb and the inactive LT A subunit (A72A), were expressed in G58-1 cells under 0.5% L-arabinose induction with a pBAD plasmid. ^**^P < 0.01 vs. the control group.

To determine whether LT has additional pathological effects on intestinal cells in addition to causing electrolyte loss, we investigated the HCT-8 cell line, which is derived from human ileocecal colorectal adenocarcinoma and is frequently used to study both ETEC and *Vibrio cholerae*. The HCT-8 cells were initially exposed to various concentrations (0–500 ng/ml) of LT for 2–24 h, after which the cytotoxic effects of LT were evaluated by CCK-8 assay (Figure 1A). Notably, LT induced dose- and time-dependent decreases in HCT-8 cell viability. We next assessed whether the inhibitory effects of LT on intestinal epithelial cell growth were correlated with increases in apoptosis. After the HCT-8 cells were treated with 0, 0.5, 5, 50, 500 ng/ml LT for 16 h, apoptosis was measured by Annexin V/propidium iodide double-staining (Figures 1B,C). The total apoptosis percentage (bottom and top right quadrant) increased gradually from 6.8 to 40.7% with increasing LT concentrations. To confirm these results, we performed the above experiments using Caco-2 cells and obtained results similar to those of the experiments involving HCT-8 cells (Figure 1D).

The percentage of apoptotic HCT-8 cells increased significantly following treatment with 0.5 ng/ml LT compared with that of the control group (*P* = 0.008) and Caco-2 cells (*P* = 0.002). In addition, to verify that the apoptotic effects in the intestinal epithelial cells were not caused by contamination, which cannot be avoided during the LT purification process, a vector pEWD501 was used. pEWD501, a derivative plasmid from pEWD299, the entire region that encoded functional LT was deleted (Dallas et al., 1979). The LT purification process was duplicated in the *E. coli* K12 containing pEWD501, and the activity and concentration of the LT toxin in the purified solution was then determined using GM1-EMSA; LT was not detected. Subsequently, the serially diluted purified solution was used to treat the HCT-8 for 16 h (Figure 1C). Because the protein concentration of *E. coli* K12 (pEWD501) purified lysates was as low as 7 ng/ml, the 50 ng/ml concentrate was obtained after ultrafiltration. Thus, the results of treatment with 500 ng/ml were missed. We found that the LT-free pEWD501 purified solution (50 ng/ml) had no significant apoptotic effect on the HCT-8 cells compared with the effect observed in the control group (*P* = 0.2484), and there was no significant apoptotic effect on the HCT-8 cells among the different concentration of purified solution (*P* = 0.5935). On the other hand, the purified protein lysates from *E. coli* containing pEWD299 induced a dose-dependent decrease in viability and increased the rate of apoptosis in HCT-8 cells. To confirm these results, we performed the above experiments using Caco-2 cells and similar results were obtained (Figure 1D). This result suggested that the effect of contamination during the LT purification process on HCT-8 cell viability was not significant.

Taken together, these results indicated that LT induced HCT-8 and Caco-2 cell apoptosis in a dose-dependent manner. Furthermore, we performed TUNEL staining to detect DNA fragmentation, one of the hallmarks of apoptosis. As expected, the percentage of TUNEL-positive cells (which exhibited green fluorescence) increased significantly from 3.2 ± 1.3 to 26.6 ± 6.5% after treatment with 50 ng/ml LT for 16 h (*P* < 0.0001) (Figure 1E). In summary, our results suggest that LT has cytotoxic effects on intestinal epithelial cells *in vitro*.

To determine the extent to which LT and different enterotoxins promote HCT-8 cell apoptosis, we amplified the genes encoding several enterotoxin subunits by PCR, cloned them into a pBAD vector, and expressed them in G58-1 cells under L-arabinose induction, after which we performed ELISA to confirm the expression of the relevant enterotoxin subunits (Johnson et al., 2009). The levels of HCT-8 apoptosis induced by the toxins produced by the modified strains were quantified and then compared to the level of apoptosis induced by the parental G58-1 strain, which contained an empty pBAD plasmid. Expressing the genes encoding the enterotoxin subunits LT-A, LT-B, STa1, and STb moderately increased HCT-8 cell apoptosis in the corresponding groups compared to the control group (Figure 1E). Specifically, expressing the indicated genes (LT-A, LT-B, STa1, and STb) increased HCT-8 cell apoptosis by 2.3 ± 0.38, 1.3 ± 0.92, 4.2 ± 0.41, and 3.1 ± 0.64-fold, respectively. However, a mutation in an LT A subunit (A72A) that rendered the toxin inactive increased HCT-8 apoptosis by only 3.5 ± 0.8-fold in the corresponding group compared to the control group. In contrast, expressing the LT holotoxin G58-1 significantly increased HCT-8 apoptosis (8.2 ± 0.6-fold, *P* < 0.0001) in the corresponding group compared to the control group, inducing a fold-change that was almost as large as that induced by the prototypical human isolate ETEC H10407 (8.9 ± 0.49-fold, *P* < 0.0001). These results suggested that (Crossman et al., 2010) the LT holotoxin and the enterotoxin subunits induced apoptosis in host HCT-8 cells, (Gupta et al., 2008) the LT holotoxin is one of the important triggers of ETEC H10407-induced HCT-8 cell apoptosis, and (Haycocks et al., 2015) ADP-ribosylation activity plays an important role in LT-induced apoptosis in HCT-8 cells.

### ETEC induces LT-dependent HCT-8 cell apoptosis and ER stress activation

**Figure 2:**
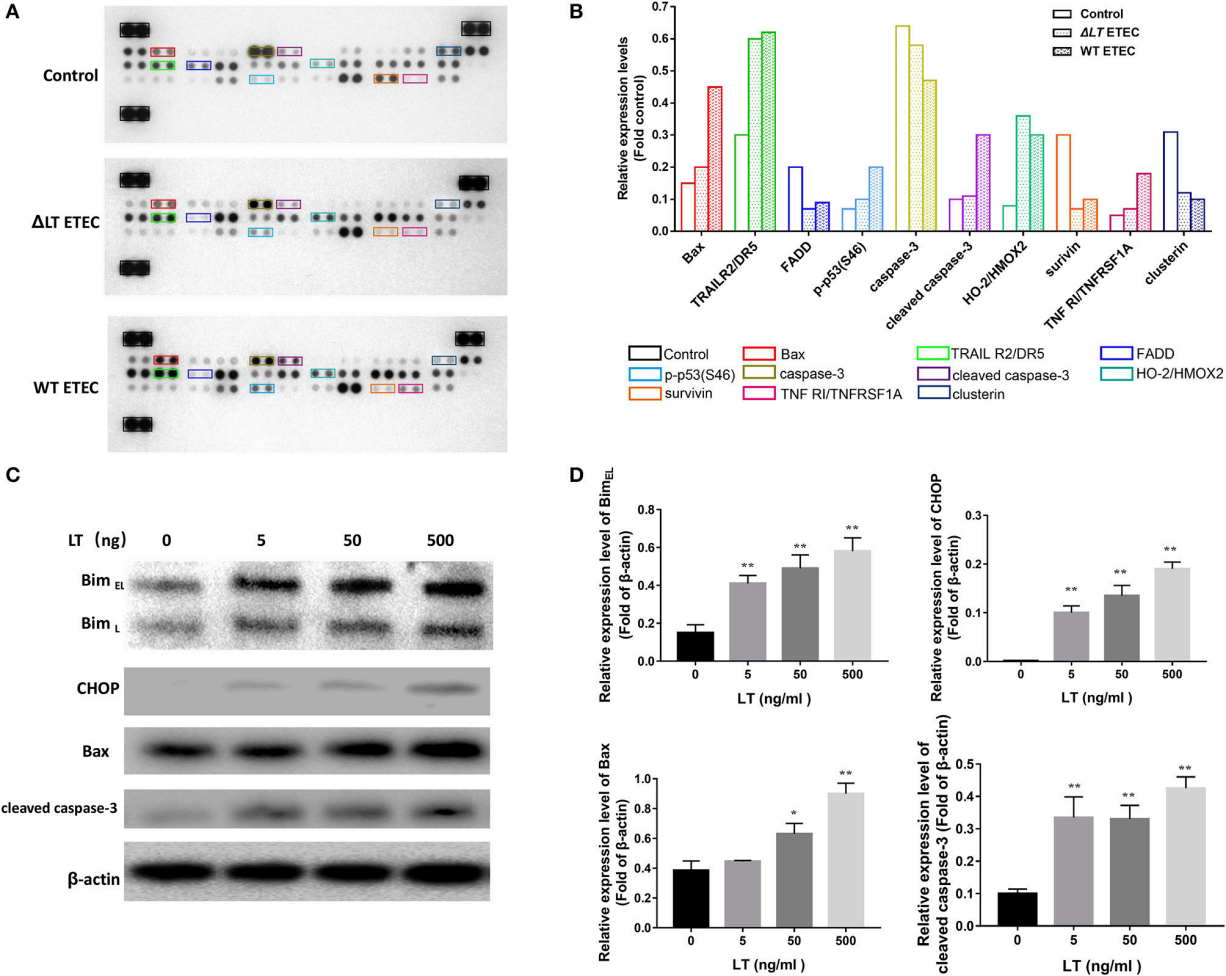
LT toxin modifies the expression of apoptosis-related proteins in HCT-8 cells. **(A)** The total-cell lysates of HCT-8 cells treated with ΔLT ETEC and wild-type ETEC, as well as those of untreated HCT-8 cells, were incubated with nitrocellulose membranes containing 35 antibodies specific for apoptosis-related proteins (Human Apoptosis Array Kit/Proteome Profiler™, R&D Systems). These differentially expressed proteins are marked by rectangles of different colors. **(B)** Bar graphs of the levels of apoptosis-related proteins whose expression changed significantly. The colors correspond to the proteins listed in panel A. **(C)** The expression levels of the proteins (Bim, CHOP, Bax, and cleaved caspase-3) that were differentially expressed in the human apoptosis array were confirmed, and the expression levels of Bim and CHOP, which are involved in ER stress-induced apoptosis, were detected. HCT-8 cells were treated with LT at different concentrations (0–500 ng/ml) for 16 h. **(D)** Bar graphs of the expression levels of the proteins in C. ^*^P < 0.05, ^**^P < 0.01 vs. the control group (β-actin).

As we showed that LT toxin inhibits cell growth and induces apoptosis in intestinal epithelial cells, we evaluated the effects of LT on the proteins involved in apoptosis and cell survival progression via protein profiler assays using a Human Apoptosis Array Kit/Proteome Profiler™ (R&D Systems). This kit analyzed the expression of 35 proteins known to be associated with the above-mentioned cellular processes. We first infected HCT-8 cells with ΔLT ETEC and wild-type ETEC and then analyzed the protein profiles of the cell lysates. Figure 2A shows that the expression levels of the following proteins were significantly different between the ΔLT ETEC-treated and untreated HCT-8 groups: TRAIL R2/DR5, FADD, HO-2/HMOX2, survivin, and Clusterin. Furthermore, the following proteins were significantly differentially expressed between the wild-type ETEC-treated HCT-8 and ΔLT ETEC-treated HCT-8 groups: Bax, p-p53(S46), cleaved caspase-3, and TNFRI/TNFRSF1A. Based on these findings, we concluded that the expression of cleaved caspase-3, an inducer of apoptosis, was up-regulated in the wild-type ETEC-treated group compared to the ΔLT ETEC-treated group. Moreover, we found that the expression of Survivin (an inhibitor of caspase-3) was down-regulated, indicating that its inhibitory effects on caspase-3 were weakened in the indicated group compared to the ΔLT ETEC-treated group. Taken together, these findings indicated that LT can promote HCT-8 cell apoptosis, and the results of this experiment indicated that LT plays an important role in intestinal epithelial cell apoptosis during ETEC infection.

According to the results of a previous study, Bax^−/−^ Bak^−/−^ mice are resistant to ER stress-induced apoptosis, indicating that Bax plays a critical role in ER stress-induced cell death (Wei et al., 2001; Ghosh et al., 2012). In this study, we found that Bax expression levels were significantly up-regulated in wild-type ETEC-treated HCT-8 cells but not in ΔLT ETEC-treated HCT-8 cells, suggesting that ER stress may be involved in LT-induced intestinal epithelial cell apoptosis. To confirm the role of ER stress in LT- mediated apoptosis, we detected the protein expression levels of Bim and CHOP in HCT-8 cells stimulated with different concentrations of LT (Figures 2C,D), as Bim plays an essential role in ER stress-induced apoptosis (Ghosh et al., 2012) and ER stress activates Bim through CHOP-mediated direct transcriptional induction (Wang et al., 1998). We found that the expression of Bim, as well as that of CHOP, was significantly up-regulated under 5 ng/ml LT toxin stimulation (*P* < 0.01). These data suggested that ER stress may participate in LT-induced intestinal epithelial cell apoptosis.

### LT activates ER stress in intestinal epithelial cells

**Figure 3:**
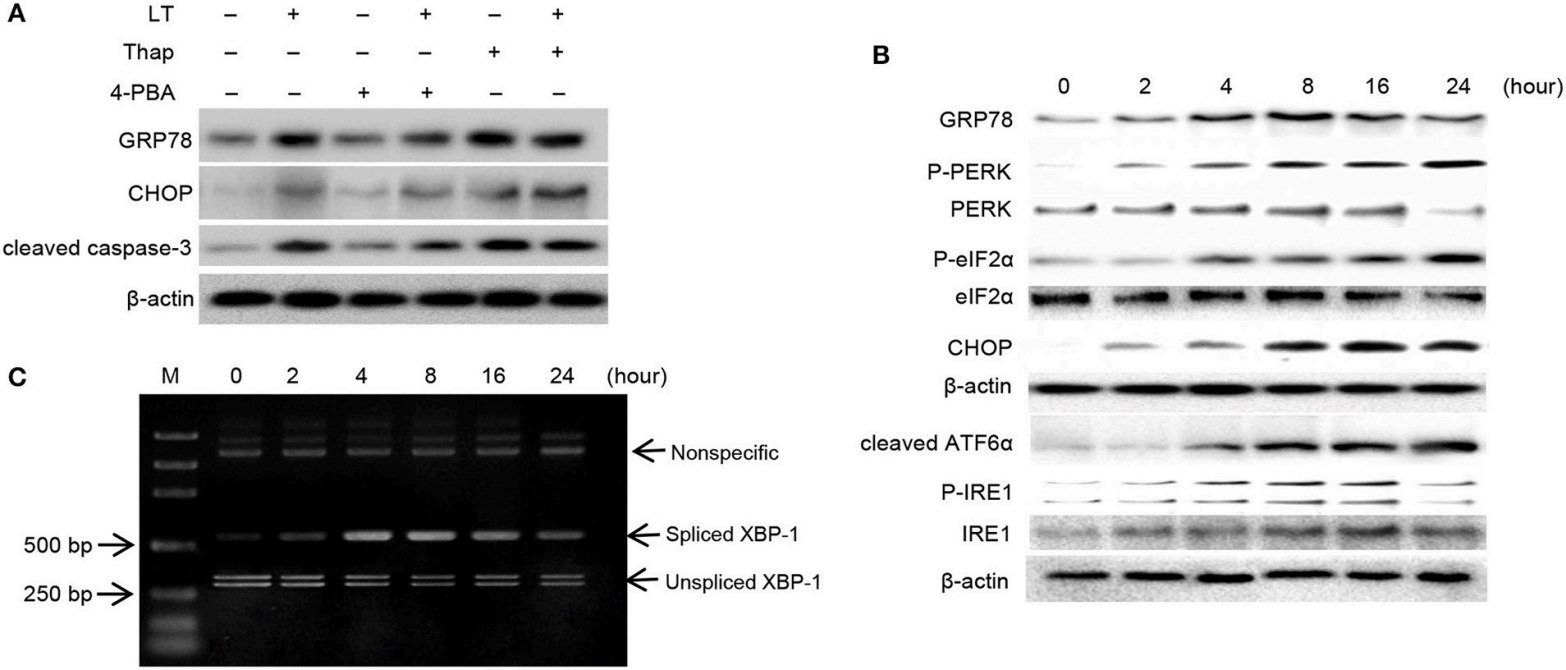
LT induces ER stress and the UPR. **(A)** The effects of ER stress inhibitors or activators on LT-induced ER stress in intestinal epithelial cells. After the cells were pretreated with 2 mM 4-PBA or 150 nM Thap for 2 h and then treated with 50 ng/ml LT for 16 h, we analyzed the expression of GRP78, CHOP, cleaved caspase-3 and β-actin by western blotting. **(B)** The effects of LT on ER stress-related proteins were time dependent. HCT-8 cells were treated with 50 ng/ml LT for 0-24 h. **(C)** LT-induced alternative splicing of XBP1 mRNA was examined by RT-PCR. The data shown in A, B, and C are from three separate experiments whose results were similar.

GRP78 and CHOP are considered to play vital roles in the ER stress response. To confirm the role of ER stress in LT-induced cell death, we pretreated HCT-8 cells with 2 mM 4-PBA and 150 nM Thap for 2 h before treating them with 50 ng/ml LT and examining the expression levels of GRP78 and CHOP, as well as those of the apoptosis executor cleaved caspase-3 (Figure 3A). We found that the ER stress inhibitor 4-PBA significantly attenuated LT-induced cell growth inhibition; however, the ER stress inducer Thap significantly promoted LT-induced cell growth inhibition and apoptosis. These results suggest that ER stress signaling pathway activation is closely related to LT-induced cell growth inhibition and apoptosis in intestinal epithelial cells.

In pathological states, the accumulation of misfolded or unfolded proteins can trigger ER stress, which is characterized by adaptive increases in the expression of ER stress-related molecules, including GRP78, p-PERK, p-eIF2α, and cleaved ATF6α. In this study, we determined the effects of LT on these transducers. We found that GRP78 expression levels increased in time-dependent manner up to 8 h after treatment and then declined slightly. Moreover, we found that p-PERK, p-eIF2α, and cleaved ATF6α expression levels, as well as CHOP expression levels, increased in a time-dependent manner (Figure 3B). p-IRE1 expression levels increased gradually within the first 16 h and then declined within the next 8 h after treatment. In addition, the expression of spliced XBP-1 mRNA, a sequence-specific substrate cleaved by p-IRE1, was determined by RT-PCR (Figure 3C). Consistent with the results pertaining to p-IRE1, the PCR results showed that the spliced form of XBP-1 mRNA was generated after treatment with LT for 4 h and that the levels of this mRNA decreased after 16 h. These findings suggest that the LT-induced IRE1 pathway was activated temporarily. Taken together, these results suggest that LT can activate the three ER stress-related pathways to varying degrees. All three pathways were activated in a time-dependent manner within 16 h of treatment.

### CHOP is essential for LT-induced apoptosis

**Figure 4:**
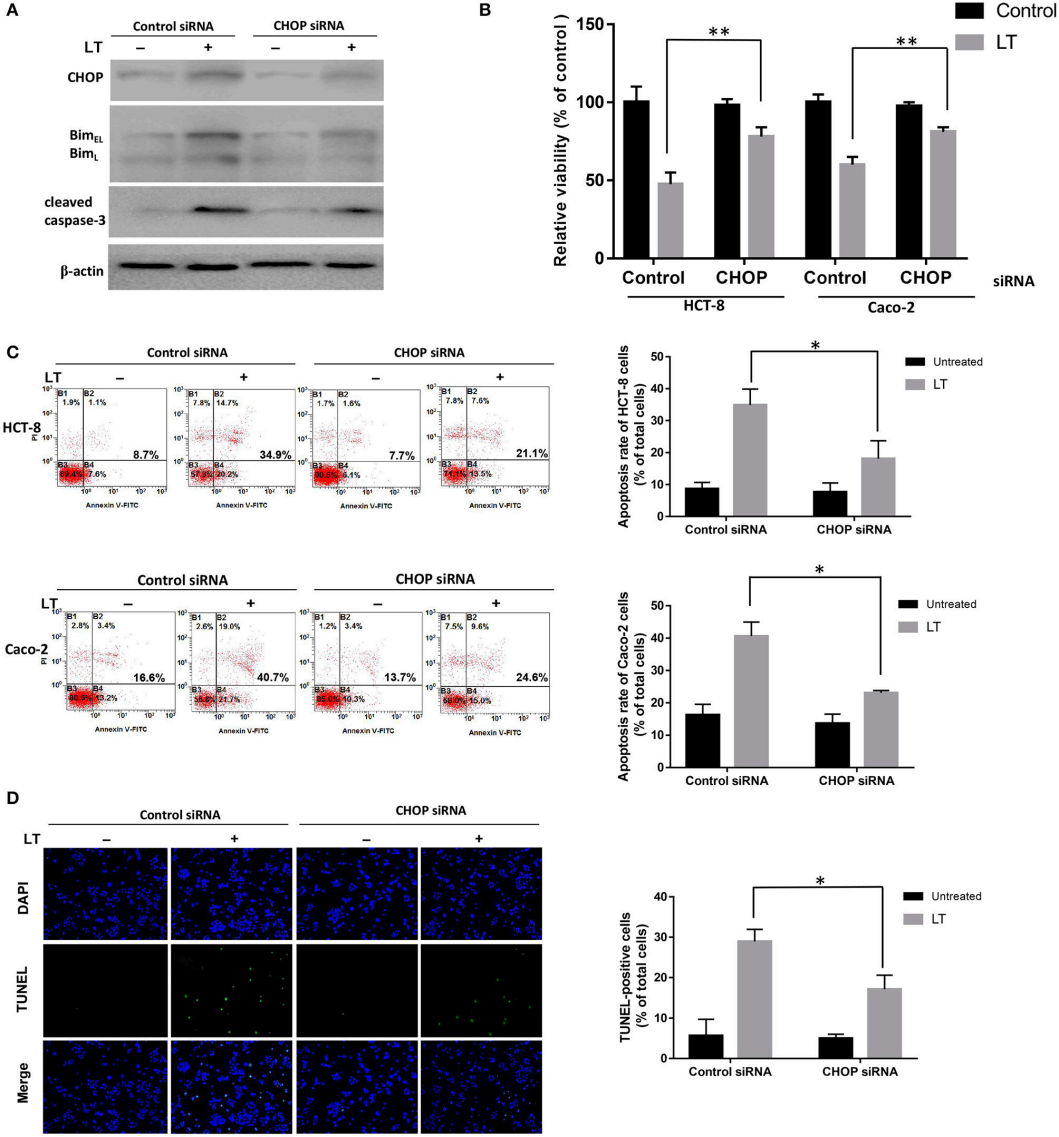
The role of CHOP in LT-induced intestinal epithelial cell apoptosis. HCT-8 and Caco-2 cells were transfected with CHOP or control siRNA (non-targeting siRNA). The expression levels of the apoptosis-related proteins and phenotypes were analyzed after the cells were treated with 50 ng/ml LT for 16 h, **(A)** CHOP, Bim, cleaved caspase-3 and β-actin expression levels in HCT-8 cells were measured by western blotting using specific antibodies. The experiment was performed in triplicate. **(B)** CCK-8 assay was used to test HCT-8 and Caco-2 cell viability. **(C)** Annexin V/propidium iodide double-staining and flow cytometric analysis were conducted to test HCT-8 and Caco-2 cell apoptosis. **(D)** TUNEL- and DAPI-strained HCT-8 cells were observed under a fluorescence microscope, and the cells displaying green and blue fluorescence, respectively, were counted. The data in **(B–D)** were the mean ± *SD* of three independent experiments. ^*^P < 0.05 vs. the control group.

All three ER stress-associated pathways, which are regulated by p-PERK, ATF6, and IRE1, respectively, can activate CHOP, which plays a significant pro-apoptotic role during ER stress. To confirm the effects of CHOP in LT-induced apoptosis in intestinal epithelial cells, we used siRNA to knock down CHOP. As shown in Figure 4A, CHOP siRNA down-regulated CHOP expression. Additionally, Bim and cleaved caspase-3 expression levels were also down-regulated after LT treatment in the corresponding group compared to the control siRNA group. HCT-8 cell viability was significantly increased (*P* = 0.0053) in the CHOP siRNA group (78.0 ± 6.0%) compared to the control group (47.5 ± 7.5%), as determined by CKK-8 assay, after 16 h of treatment with 50 ng/ml LT (Figure 4B). The effect of CHOP knockdown on HCT-8 cell apoptosis was measured by Annexin V/propidium iodide-double staining, and the results showed that LT significantly induced apoptosis in 34.9 ± 5.0% of cells in the control group and only 18.1 ± 5.6% of cells in the CHOP-knockdown group (Figure 4C) (*P* = 0.0181). TUNEL staining showed that the percentage of TUNEL-positive HCT-8 cells decreased from 28.9 ± 3.0% in the control group to 17.1 ± 3.4% in the CHOP-knockdown group (*P* = 0.0111) (Figure 4D). To confirm these results, we performed the above experiments using Caco-2 cells and obtained results similar to those of the experiments involving HCT-8 cells. Specifically, we noted that Caco-2 cell viability increased from 60.2 ± 5.0% in the control group to 80.8 ± 3.1% in the CHOP-knockdown group (*P* = 0.0034). LT induced apoptosis in 40.6 ± 4.3% of cells in the control group and only 23.1 ± 0.7% of cells in the CHOP-knockdown group (*P* = 0.0296).

### The PERK-CHOP pathway is mainly involved in ER stress-mediated apoptosis in intestinal epithelial cells treated with LT

**Figure 5:**
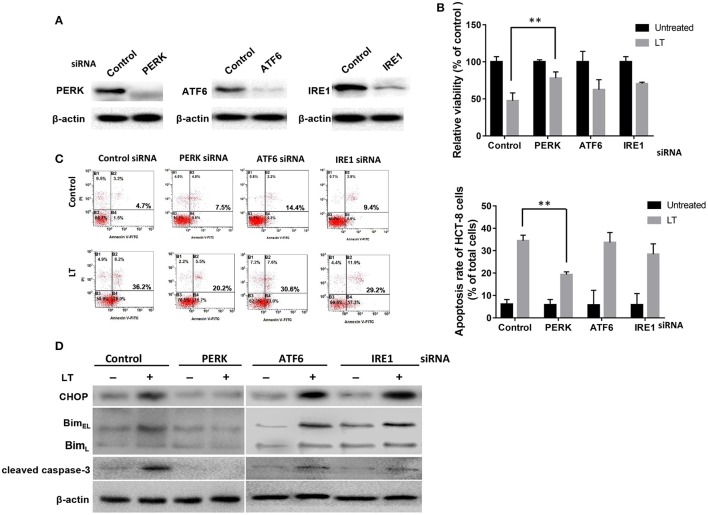
The role of the three UPR pathways in LT-induced apoptosis in intestinal epithelial cells. **(A)** We assessed the inhibition of efficiency of siRNAs targeting PERK, ATF6 and IRE1 by transfecting HCT-8 cells with the indicated siRNAs at the indicated concentration (100 nM of each) for 24 h and determining the expression levels of the target proteins by western blotting. **(B)** The transfected cells were treated with 50 ng/ml LT for 16 h, after which cell viability was measured by CKK-8 assay. **(C)** The corresponding apoptosis rates were determined by Annexin V/propidium iodide double-staining and are shown in the bar graph. **(D)** The effects of PERK, ATF6 or IRE1 knockdown on LT-induced ER stress-related protein expression. The gel results are from experiments performed in triplicate whose results were similar. The bar graph data shown in **(C,D)** represent the mean ± SD from three independent tests. ^*^P < 0.05, ^**^P < 0.01 vs. the control group.

All three arms of the ER stress signaling pathway, which are initiated by ATF6, IRE1α, and PERK, respectively, are capable of inducing CHOP, which plays an important role in ER stress-mediated apoptosis. Different bacterial factors activate ER stress through different pathways. Some of these factors, such as LPS, Stx1, and LT (in this study), induce all three ER stress axes, while some factors have more specific effects. For example, ESAT-6 induced XBP1-S and eIF2α activation in A549 cells (Choi et al., 2010), whereas aerolysin only induced XBP1-S activation (Bischof et al., 2008). To determine which pathway plays a key role in LT-induced ER stress-related apoptosis, we knocked down the expression of the three transducers with specific siRNAs (Figure 5A). CCK-8 was used to analyze the viability of HCT-8 cells incubated with 50 ng/ml LT for 16 h. The results showed that HCT-8 cell viability was significantly increased (*P* = 0.0053) in the PERK-knockdown group (47.5 ± 7.5%) compared to the control group (78.1 ± 6.0%) (Figure 5B). As expected, HCT-8 apoptosis was significantly decreased (19.3 ± 1.3%) compared to that in the control group (5.9 ± 2.3%, *P* = 0.0090) (Figure 5C). Moreover, CHOP, Bim, and cleaved caspase-3 expression was not detected in the PERK-knockdown group (Figure 5D). These data indicated that the PERK-CHOP pathway rather than the other two pathways participates in LT-induced apoptosis.

### The relationship between LT-induced ER stress and ROS

**Figure 6:**
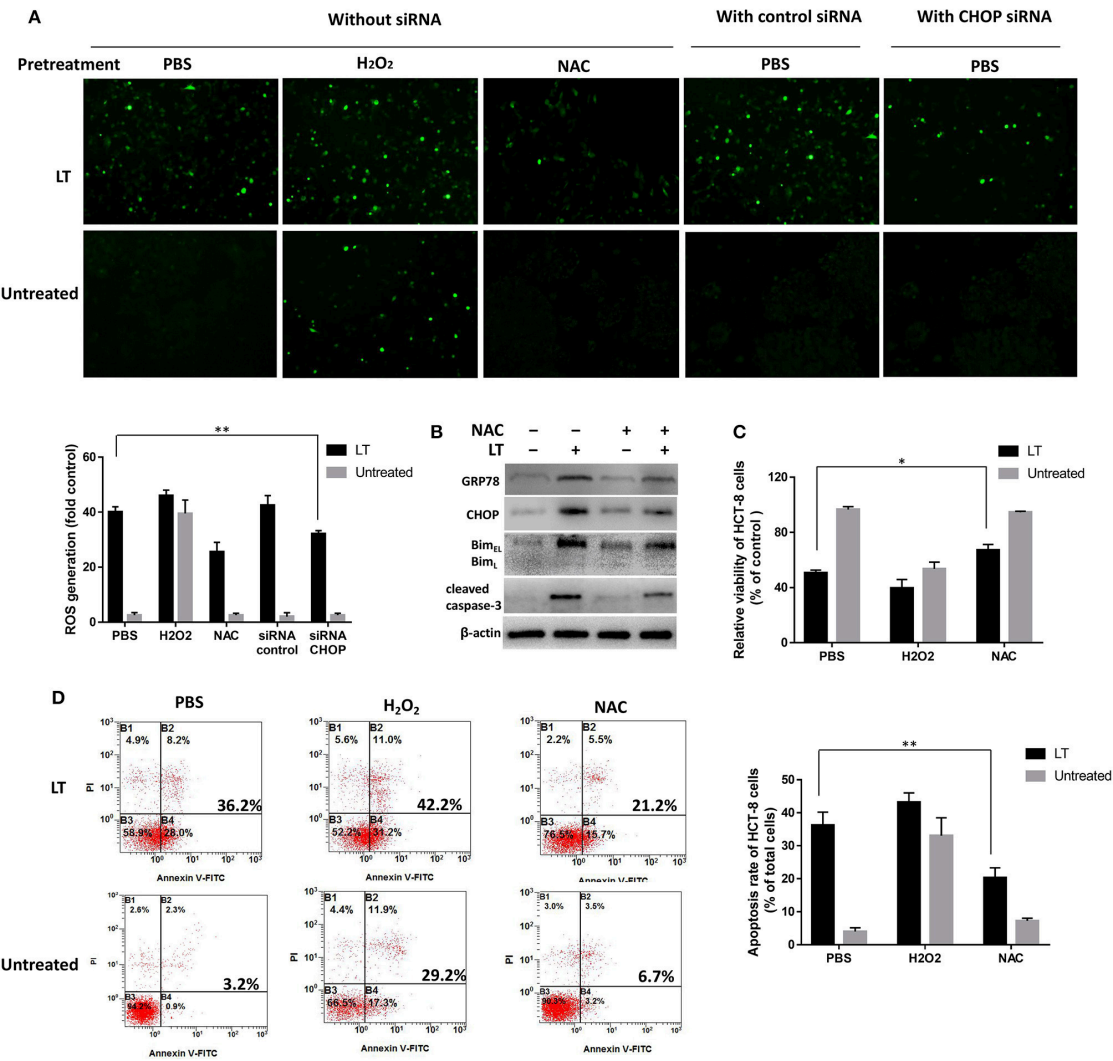
Mutual promotion-inducing effects of ROS production and ER stress induced by LT in HCT-8 cells. HCT-8 cells were pretreated with PBS (as a control), H_2_O_2_, and 10 nM NAC for 2 h and then treated with 50 ng/ml LT for 16 h. **(A)** ROS levels were detected by DCFH-DA and observed by fluorescence microscopy. HCT-8 cells were also treated with CHOP siRNA and control siRNA. The mean DCFH fluorescence intensity, an indicator of ROS generation, was measured by flow cytometry, and the fold changes in fluorescence intensity in the indicated groups relative to the control group (PBS) are shown in the histogram under the fluorescent images. **(B)** The effects of NAC on the expression of the LT-induced ER stress-related proteins GRP78, CHOP, Bim, cleaved caspase-3, and β-actin (loading control) were analyzed. **(C)** HCT-8 cell viability was examined by CCK-8 assay. **(D)** HCT-8 cell apoptosis was analyzed by Annexin V/propidium iodide double-staining followed by flow cytometry. The gel results are from experiments performed in triplicate whose results were similar. The bar graph data shown in panels **(C,D)** represent the mean ± SD from three independent tests. ^*^P < 0.05 vs. the control group (without LT treatment or PBS pretreatment).

Intestinal epithelial cells infected by microbial pathogens typically activate Rho GTPases to produce ROS to kill bacteria; however, prolonged ROS generation has been implicated in DNA damage. In this study, to investigate the relationship between ROS production and LT-induced ER stress-related apoptosis, we first investigated the effect of LT on ROS production in intestinal epithelial cells. We used the ROS scavenger NAC and the accelerant H_2_O_2_ to block and promote ROS generation, respectively. PBS was used as a control. HCT-8 cells were pretreated with the aforementioned ROS scavenger or accelerant for 2 h before being subjected to treatment with 50 ng/ml LT for 16 h. Finally, ROS levels were detected by DCFH-DA and observed by fluorescence microscopy. PBS pretreatment increased ROS levels significantly in the LT treatment group compared to the untreated group (*P* < 0.001) (Figure 6A, without siRNA). In addition, ROS levels were increased by H_2_O_2_pretreatment and decreased by NAC pretreatment. These results showed that LT can promote ROS production in HCT-8 cells.

Then, we tested the effects of inhibiting ROS production on ER stress and examined the expression levels of the ER-stress-related proteins GRP78, CHOP, Bim, and cleaved caspase-3 after the HCT-8 cells were treated as mentioned above. In addition, HCT-8 cell viability and apoptosis were assessed by CCK-8 and flow cytometry assay. LT-induced GRP78 and CHOP expression, as well as LT-induced Bim and cleaved caspase-3 expression, was attenuated in the NAC-pretreated group compared to the control group (Figure 6B). Moreover, cell viability was significantly (*P* = 0.0124) increased in the NAC-pretreated group (67.1 ± 3.0%) compared to the control group (55.5 ± 3.4%) (Figure 6C). LT treatment promoted apoptosis, an effect that was inhibited by NAC to a certain extent (Figure 6D). Specifically, the rate of LT-induced apoptosis was significantly decreased from 36.2 ± 4.0% in the PBS pretreatment group to 20.3 ± 3.1% in the NAC pretreatment group (*P* = 0.0054). These results indicated that NAC-mediated reductions in ROS accumulation can relieve ER stress and restore cell viability in intestinal epithelial cells.

We also examined whether ER stress could regulate LT-induced ROS production. We examined ROS production after siRNA-mediated CHOP knockdown in LT-treated HCT-8 cells. LT-induced ROS production was significantly reduced by CHOP knockdown in HCT-8 cells, from 40.1 ± 1.9 to 32.1 ± 1.2% (*P* = 0.0034) (Figure 6A, with siRNA), suggesting that blocking ER stress can suppress LT-induced ROS production. These data indicated that ROS production and ER stress have mutual promotion-inducing effects in LT-induced HCT-8 cells.

### Effect of inhibiting ROS generation on LT-induced apoptosis in the small intestinal mucosa of ETEC inoculated mice

**Figure 7:**
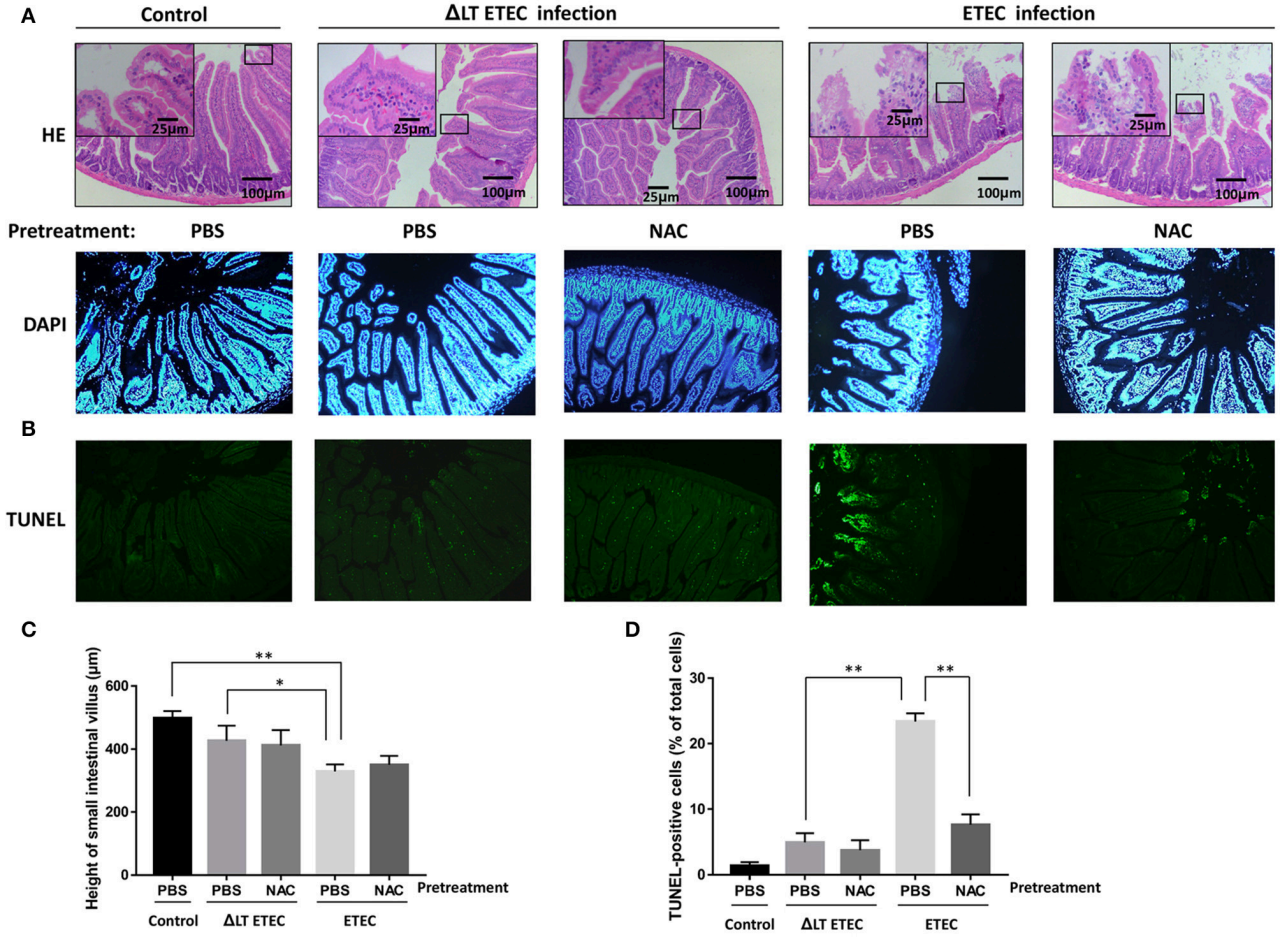
Effect of NAC on LT-induced apoptosis in the small intestinal mucosa (mice). First, PBS (control) or NAC (ROS scavenger) was intraperitoneally administered to the mice 1–3 h prior to ETEC or ΔLT ETEC inoculation. Then, the mice in the ETEC or ΔLT ETEC infection group were orally inoculated with a suspension of ETEC (2*10^7^ cells). **(A)** Light micrographs of the small intestine tissues, which were stained by HE (×200). **(B)** TUNEL- and DAPI-stained small intestinal mucosal sections pretreated with or without NAC before being inoculated with ETEC were observed under a fluorescence microscope. **(C)** The bar graphs of the small intestinal villus height in ETEC or ΔLT ETEC infected mice with or without NAC pretreatment. **(D)** Bar graphs of the number of TUNEL-positive cells and the percentage of DAPI-positive cells, which represent the mean ± SD from three independent tests. ^*^P < 0.05, ^**^P < 0.01, vs. the control group.

To investigate whether inhibiting ROS production can attenuate LT-induced apoptosis during ETEC infection *in vivo*, we established the ETEC infection model. In the ΔLT ETEC infection group, intestinal edema was observed, but the destruction of the small intestine villus tips was not obvious (Figures 7A,C). With the PBS pretreatment, there was no significant difference in the small intestinal villus length between the ΔLT ETEC infection group (426.7 ± 47.5 μm) and the control group (498.3 ± 22.5 μm) (*P* = 0.0776) or between NAC pretreatment (411.7 ± 49.2 μm) and PBS pretreatment (*P* = 0.8101) (Figures 7A,C). The percentage of TUNEL-positive cells was 4.9 ± 1.4% with PBS pretreatment and 3.7 ± 1.5% with NAC pretreatment, which was not significantly different (*P* = 0.4661) (Figures 7B,D).

In the ETEC infection group, intestinal edema was observed, and the integrity of small intestine villus tips was obviously damaged (Figure 7A). With the PBS pretreatment, the height of small intestinal villi (329.3 ± 22.2 μm) was significantly shorter than that in the control group (*P* = 0.0008) and the ΔLT ETEC infection group (*P* = 0.0325). Furthermore, with the PBS pretreatment, the number of TUNEL-positive cells in the ETEC infection group (23.4 ± 1.3%) was significantly increased compared to that of the ΔLT ETEC infection group (*P* < 0.0001). With the NAC pretreatment, the villus height (350.3 ± 28.5 μm) did not significantly recover (*P* = 0.3498) and the destruction of the integrity of the villus tips was not alleviated (Figures 7A,C), but the number of TUNEL-positive cells was significantly reduced from 23.4 ± 1.3% with the PBS pretreatment to 7.6 ± 1.6% with the NAC pretreatment (*P* = 0.0097) (Figures 7B,D).

## Discussion

LT is a key ETEC virulence factor, and previous studies have shown that LT is involved in a variety of processes, such as the adherence of ETEC to host cells (Wijemanne and Moxley, 2014). In addition, LT can even promote the intestinal colonization of *Salmonella enterica* (Verbrugghe et al., 2015). In addition, LT increases fluid secretion and cooperates with ST to alter cyclic nucleotide production in intestinal epithelial cells (Read et al., 2014). Based on the classical definition, enterotoxin stimulates net secretion in ligated intestinal segments without histological evidence of intestinal damage or evidence of injury to non-erythrocyte cells in *in vitro* assays. However, in this study, we found that LT can trigger decreases in intestinal epithelial cell viability and induce apoptosis in a dose- and time- dependent manner in both HCT-8 and Caco-2 cells. The signaling pathway involved in these processes has been investigated.

First, we determined the effects of LT and ETEC on the proteins involved in intestinal epithelial cell apoptosis with protein arrays. Although the apoptotic effects caused by potential contamination during LT purification process was exclude in the cytology experiment, however during the actual infection process, pathogen-associated molecular patterns (PAMPs) may also play an promoting role in apoptosis such as LPS and STa, which should be taken into consideration. In this study to simulate the process of ETEC infection in intestinal epithelial cells, we treated HCT-8 cells with wild-type ETEC and ΔLT ETEC. We found that Bax, p-p53(S46), cleaved caspase-3, and TNFRI/TNFRSF1A expression levels were significantly up-regulated in wild-type ETEC- but not in ΔLT ETEC-infected HCT-8 cells (Figures 2A–C), indicating that these proteins may be involved in LT-induced intestinal epithelial cell apoptosis during ETEC infection. These data may reflect interactions between LT and other ETEC pathogenic factors, (ST and LPS), but they showed that LT plays a key role in cell apoptosis. Furthermore, we found LT alone can lead to Bim, Bax, and cleaved caspase-3 upregulation (Figures 2C,D). A previous study showed that Bax and Bak homooligomerization may result in the release of cytochrome c from mitochondria (Adams and Cory, 1998; Kroemer and Reed, 2000). Bax- and Bak-double-deficient cells can resist apoptosis induced by ER stress; however, Bak-deficient cells cannot (Wei et al., 2001). This result suggests that Bax is essential for ER stress-induced apoptosis (Ghosh et al., 2012). In this study, Bax was significantly up-regulated in wild-type ETEC-, but not in ΔLT ETEC-infected HCT-8 cells, suggesting that ER stress may play an important role in LT-induced apoptosis. To confirm this hypothesis, we explored the role of ER stress in LT-induced intestinal epithelial cell apoptosis.

Many bacterial effector proteins, including toxins, such as Stx, SubAB, or PFTs, can modulate host cell transcription and translation processes, including protein biosynthesis. These phenomena are critically important for indirect microbe detection or even targeted therapy (Szegezdi et al., 2006). ER stress is a cell response designed to balance the accumulation of unfolded proteins in the ER under certain stress states. During bacterial infections, different ER stress sensors are activated, suggesting that bacteria have evolved strategies for inducing a particular UPR pathway. *Shigella dysenteriae* 1 activates Sx1, and enterohemorrhagic *E. coli* activates PERK and ATF6 but not IRE1 in THP-1 cells (Lee et al., 2008). In this study, we showed that LT can induce up-regulation of GRP78, the master regulator of these pathways, in HCT-8 cells, as well as p-PERK, cleaved-ATF6, p-IRE1. These results suggested that all three ER stress-related signaling pathways are initiated during this process. Furthermore, siRNAs were used to knock down the expression of the three transducers independently; however, the rate of LT-induced apoptosis decreased significantly in only the PERK-knockdown group. Cleaved caspase-3 was down-regulated in the indicated group. This result indicated that the PERK pathway plays a substantial role in LT-induced apoptosis. Activated PERK initially blocks general protein synthesis and aids cell survival by decreasing the loads of nascent proteins arriving in the ER by phosphorylating eIF2α and ATF4 (Szegezdi et al., 2006); however, PERK activation also leads to the induction of CHOP, an important component of the switch from pro-survival to pro-apoptotic signaling whose induction depends strongly on ATF4, a downstream effector of the PERK signaling pathway (Szegezdi et al., 2006).

ATF6 was activated in LT-treated HCT-8 cells; however, the RNA interference test results showed that ATF6 was not involved in LT-induced apoptosis. Evidence indicates that ATF6 can induce CHOP expression, but no reports have linked ATF6 to ER-stress induced apoptosis (Szegezdi et al., 2006). IRE1 has pro- and anti-apoptotic functions. These functions are regulated by c-Jun N-terminal inhibitory kinase (JIK), which could bind both IRE1 and TNF-receptor-associated factor 2 (TRAF2) (Yoneda et al., 2001; Oono et al., 2004). The IRE1-TRAF2 complex can recruit apoptosis-signal-regulating kinase (ASK1), which could relay various stress signals to JNK and p38 (Nishitoh et al., 1998) In this study, TNF R1, an upstream protein of TRAF2, was expressed at higher levels in wild-type ETEC-infected cells than in ΔLT ETEC-infected cells and control cells (Figures 2A,B). However, IRE1 knockdown did not attenuate the rate of LT-induced apoptosis, perhaps because the apoptotic signal was not passed to JNK or p38 (additional experiments are needed to confirm this issue). The findings of this study suggested that IRE1 is not essential for LT-induced apoptosis.

It is worth noting that some apoptosis-related proteins were expressed at high levels in the ΔLT ETEC infection group compared to the control group and that these proteins were expressed at even higher levels in the wild-type ETEC infection group than in the control group (Figures 2A,B). These proteins included TNF-related apoptosis inducing ligand receptor 2/Death receptor 5 (TRAIL R2/DR5), which participates in an extrinsic pathway that induces apoptosis via pro-caspase-8 activation (Macewan, 2008; Zaitseva et al., 2011) These data suggested that ETEC contains other factors that induce intestinal epithelial cell apoptosis in addition to LT toxin. Evidence shows that lipopolysaccharide (LPS) stimulated apoptosis through TNFR1 but not TNFR2 (Alikhani et al., 2004; Kwon et al., 2006). In addition, our data showed that Sta1, which is encoded by the *sta1* gene in the ETEC H10407 plasmid, can increase HCT-8 cell apoptosis by 4.2-fold (Figure 1F). However, whether TRAIL R2/DR5 plays a key role in Sta1-induced apoptosis requires further study.

ROS act as important multi-functional signaling molecules that regulate several cellular pathways and play a key role in cell fate determination (Pierre et al., 2013; Zhou et al., 2014). In this study, exposing intestinal epithelial cells to LT caused significant increases in ROS production and apoptosis. We therefore sought to assess whether LT-induced apoptosis is also dependent on ROS generation and explored the relationship between ROS generation and ER stress. We found that pretreatment with antioxidants (NAC) could inhibit the expression of GRP78, CHOP, Bim, and cleaved caspase-3 and decrease the rate of apoptosis in HCT-8 cells. However, we also found that knocking down CHOP expression could reduce ROS production. A previous study showed that the PERK pathway is involved in ROS-mediated ER stress (Verfaillie et al., 2012), a finding consistent with those of this study. The *in vivo* experiments showed that NAC could not completely restore the height and integrity of small intestinal villi; however, it could significantly attenuate cell apoptosis. In additional, it should be noted that the enterocytes and underlying lamina propria was separated in the villous tips of control group (Figure 7A), a mild post-mortem change occurs only within minutes after death. It is different from the damage caused by ROS induced changes in ETEC infection group.

In conclusion, the results of this study indicate that the ER stress response is involved in LT-induced intestinal epithelial cell apoptosis during ETEC infection, a process that was partially mediated by ROS generation. NAC could alleviate LT-induced intestinal mucosal cell apoptosis in the ETEC infection mouse model. These findings may improve the understanding of the relationship between LT and the host.

## Author contributions

XL was responsible for experiment design and paper writing; CML and PL were responsible for cell culture and vector construction; CCL was responsible for apoptosis and ROS detection as well as western blot analysis; EF was responsible for PCR and siRNA experiment; YX and FJ were responsible for thesis modification.

### Conflict of interest statement

The authors declare that the research was conducted in the absence of any commercial or financial relationships that could be construed as a potential conflict of interest.
